# Diuretic inhibition of experimental myopia implicates retinal ion-driven efflux in the regulation of ocular growth

**DOI:** 10.3389/fmed.2026.1781955

**Published:** 2026-05-11

**Authors:** Melanie J. Murphy, Nina Riddell, David P. Crewther, Brian M. Ignacio, Sheila G. Crewther

**Affiliations:** 1 School of Psychological Science, La Trobe University, Melbourne, VIC, Australia; 2 Centre for Human Psychopharmacology, Swinburne University of Technology, Melbourne, VIC, Australia

**Keywords:** chick model, defocus, diuretics, electroretinogram, ENaC (epithelial Na+ channel), KCC cotransport, myopia, NKCC cotransport

## Abstract

**Introduction:**

Myopia (short-sightedness), characterized by excessive ocular growth, is the most common visual disorder and is the greatest risk factor for blindness later in life, though its etiology is uncertain. We have previously suggested that ocular volume regulation is related to the rate of Retinal Ion-Driven fluid Efflux from the vitreous to the lymphatic sinusoids of the choroid, and predict that common clinically approved diuretics (amiloride, bumetanide, and furosemide) known to act on different ion exchanger and cation-chloride symporter mechanisms in retina and RPE would show differential, sign-dependent effects on refractive compensation to optical defocusing lenses that normally induce myopia and hyperopia (long-sightedness) in chick.

**Methods:**

On day 5, 169 post-hatching chicks were intravitreally injected with 5 μL of either 1 mM amiloride (a potassium sparing diuretic that blocks sodium channels), bumetanide or furosemide (both loop diuretics that predominantly inhibit Na^+^/2Cl^–^/K^+^ co-transporters) in DMSO vehicle, or DMSO carrier control only, and fitted with + 10D, -10D lens, or No Lens. After 4 days of rearing, biometric data were collected. Electroretinograms (ERGs; *N* = 3–4 per drug group) were recorded in No Lens animals to assess the effects of diuretics on retinal light responses, both acutely (day 5) and 96 h post-injection (day 9).

**Results:**

Furosemide and amiloride reduced myopia development following negative lenses, while bumetanide and amiloride suppressed hyperopia development in response to positive lenses. ERG waveforms demonstrated that retinal integrity was maintained following drug injection, with evidence of modulation of the strength of ON vs. OFF pathway signaling compared with DMSO.

**Discussion:**

Thus, diuretics that alter retinal function and associated ion-driven transretinal/RPE fluid efflux from vitreous to choroid inhibit the induction of defocus-induced refractive errors. This highlights the potential of diuretic-like agents for the therapeutic management of myopia and associated myopia-induced ocular pathology.

## 1 Introduction

Myopia (short-sightedness) and hyperopia (longsightedness) are the most common visual disorders worldwide, with prevalence and severity of myopia increasing dramatically, and earlier onset associated with advancing urbanization, education and technology (1–3). Fifteen to 20% of myopia patients are likely to develop severe sight threatening pathologies such as glaucoma, retinal maculopathy and retinal detachment in later life (4–7). Thus, identification of classes of therapeutics targeted at controlling the axial elongation of myopic eyes is crucial if the more severe secondary complications associated with extreme myopia are to be limited and managed.

Myopia occurs when the axial length and optical components of the eye result in images focusing in the vitreous chamber rather than on the retina. Experimental myopia can be modeled in animals using monocular occlusion, or negative defocusing lenses to induce excessive ocular growth and trigger refractive compensation. Gross ocular volume is controlled by a balance between the fluid production of the ciliary body and efflux from the anterior chamber via Schlemm’s Canal, and posteriorly via transretinal fluid movements from vitreous to the choroid (8). Such efflux is driven via the polarized membranes of epithelial cells of the ciliary body (9–11) and retinal pigment epithelium (RPE). Indeed, the mechanisms underlying light dark modulation, resulting in ion transport, and altered subretinal space (SRS) and RPE volume as elaborated by Gallemore et al. (12), Steinberg and others (13–20), are the basis of the ‘Retinal Ion Driven Efflux (RIDE) Model of myopia (21). The RIDE model posits that interactions between outer retinal cellular activity and the ionic microenvironment of the SRS and RPE, including the osmoregulation of Cl^–^, Na^+^, and K^+^ transport, as well as water transport via aquaporin (AQP) channels expressed across retinal cell types (13, 22, 23) determines the rate of transretinal fluid outflow to provide a key mechanism driving rapid ocular growth in response to optical defocus.

The importance of ion and fluid transport mechanisms in ocular refractive error development is supported by a growing body of discovery-driven molecular studies in both humans and animal models. Human genome-wide association studies have consistently shown that loci associated with refractive error are enriched for ion channel and transporter activity, particularly involving cation transport (24–26), and that these loci exert their influence on refractive development early in life (26). Importantly, different developmental trajectories of ion and solute transport protein expression in the retina/RPE (including those related to GABA signaling) differentiate lens-induced myopia and hyperopia models as refractive compensation to lenses progresses in animal models (27, 28).

Further support for a role of ion-mediated signaling in myopia comes from occlusion paradigms, where profound form deprivation myopia of chick retina is associated with suppression of ligand-gated chloride transport pathways via down-regulation of glycine and GABA ionotropic receptors (29). Similarly, transcriptomic analyses of mouse occlusion myopia have identified ion transport–related gene ontologies, particularly potassium channels, as dominant features of differentially expressed long non-coding RNAs, with additional involvement of GABA signaling pathways in circular RNAs (30). Together, these findings indicate that ion transport and inhibitory neurotransmission, both mechanisms central to retinal light processing, are repeatedly implicated across genetic, proteomic, and transcriptomic levels in refractive error development.

Despite this converging evidence, direct experimental manipulation of transretinal fluid movement as a means of controlling refractive development has been limited. To date, the only study to explicitly target myopia via modulation of ion-driven fluid transport employed the loop diuretic bumetanide, a selective inhibitor of NKCC cotransporters within the cation–chloride cotransporter (CCC) family (31), and demonstrated inhibition of refractive compensation to negative, but not positive, defocusing lenses (32). Other clinically approved diuretics have not been systematically tested for myopia control, despite substantial evidence that agents such as furosemide (a loop diuretic with broader CCC activity), amiloride (a potassium-sparing diuretic targeting sodium–hydrogen exchange) (33), and acetazolamide (a carbonic anhydrase inhibitor) antagonize fluid transport processes within the retina, retinal pigment epithelium (RPE), and ciliary body (20, 32, 34–49).

**FIGURE 1:**
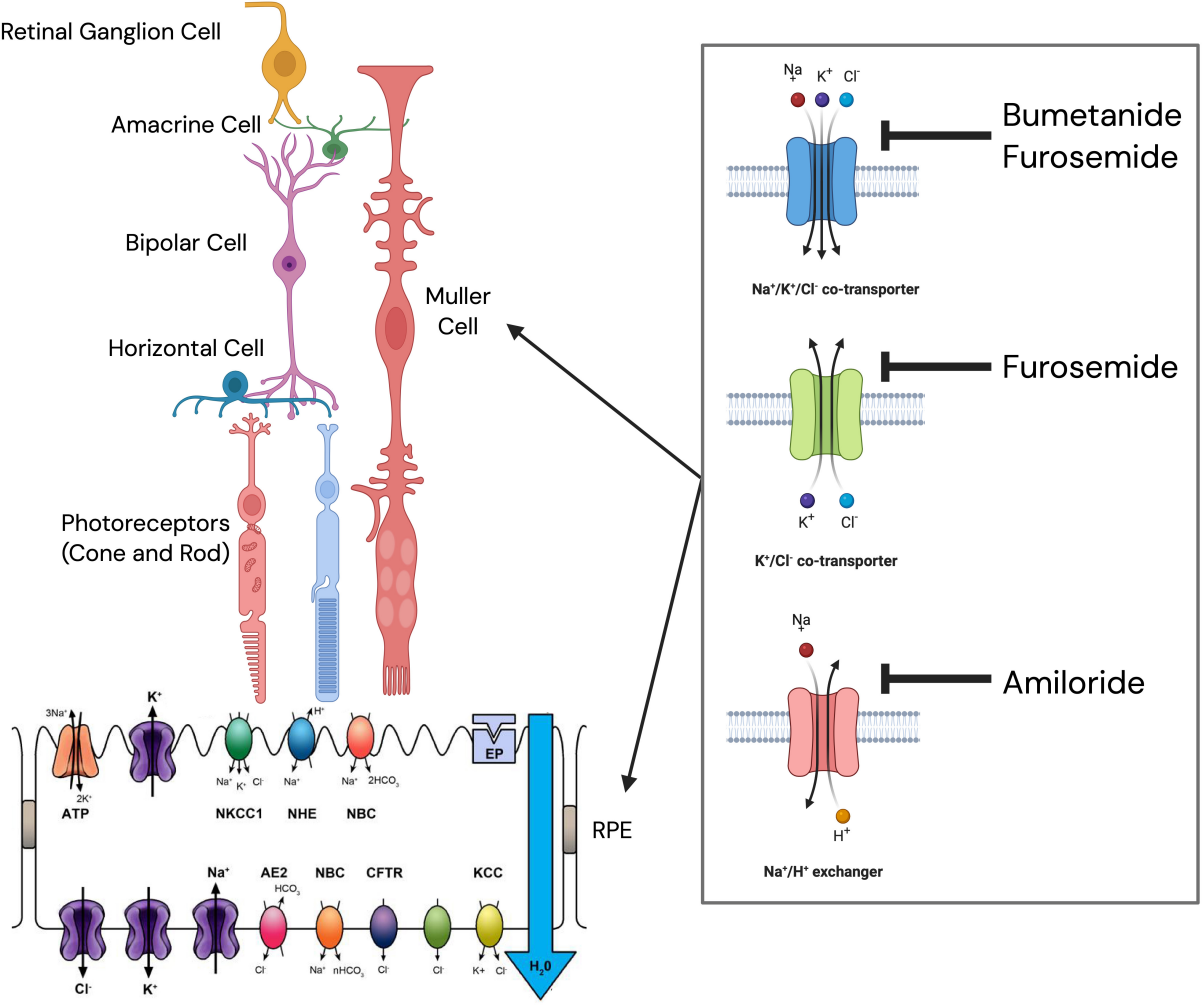
Sites of action of diuretic drugs within the retina and retinal pigment epithelium (RPE). Schematic of the neural retina and retinal pigment epithelium (RPE) showing putative sites of action of diuretic agents within Müller glial cells and across the apical (photoreceptor-facing) and basolateral (choroid-facing) membranes of the RPE. Drug-sensitive pathways include amiloride-sensitive ENaC and NHE, and bumetanide- and furosemide-sensitive NKCC and KCC, alongside key ion transport mechanisms regulating fluid and ionic homeostasis (Na^+^/K^+^-ATPase, NBC, AE2, CFTR, and K^+^/Cl^–^ channels). Epinephrine (EP) is indicated as a modulator of ion transport. In part created in https://BioRender.com. ENaC, epithelial sodium channel; NHE, Na^+^/H^+^ exchanger; NKCC, Na^+^-K^+^-2Cl^–^ cotransporter; KCC, K^+^-Cl^–^ cotransporter; Na^+^/K^+^-ATPase, sodium–potassium ATPase; NBC, sodium bicarbonate cotransporter; AE2, anion exchanger 2; CFTR, cystic fibrosis transmembrane conductance regulator; EP, epinephrine. Figure in-part created in https://BioRender.com.

The CCC family of transporters plays a central role in linking light-evoked retinal activity to ion and water flux. These ubiquitous proteins mediate electroneutral transport of chloride ions together with potassium and/or sodium, accompanied by obligate water movement (20, 50, 51). In the retina, NKCC and KCC transporters are expressed on both the RPE and retinal ON and OFF bipolar cells, where they establish distinct intracellular Cl^–^ gradients that are fundamental to depolarizing and hyperpolarizing responses to light increments and decrements, respectively (52–55). Through these mechanisms, light-driven activation of retinal ON and OFF pathways is intrinsically coupled to chloride and potassium fluxes and, by extension, to water movement across the retina and RPE, in addition to fluid flow across the ciliary body (9, 10, 56–58) (Figure 1).

Consistent with this framework, pharmacological inhibition of CCCs profoundly alters light-dependent fluid dynamics. Bumetanide, the most selective NKCC inhibitor (59, 60), has been shown to increase fluid absorption at the apical membrane of the RPE by reducing retina-to-choroid transport of K^+^ and Cl^–^ (34, 36, 61–63). In the chick, this leads to acute suppression of the light-induced increase in subretinal space volume and enhanced retinal adhesion (14, 64). Furosemide similarly affects NKCC transport but exhibits greater sensitivity for KCC-mediated pathways (65), while amiloride, through inhibition of sodium exchange, also acutely reduces light-induced subretinal space expansion (as summarized in (12, 66) and alters non-pigmented epithelium and ciliary body volume (67–69). Such findings collectively support a model in which light-dependent retinal signaling regulates transretinal fluid flow (and therefore ocular growth) via ion-driven mechanisms.

Thus, the present study aimed to test key predictions of the RIDE model, which proposes that refractive compensation to optical defocus (particularly negative defocus that induces myopia) is regulated by ion transporter–mediated fluid movement across the retina and RPE. Specifically, we examined whether pharmacological manipulation of membrane-bound ion cotransporters using clinically approved diuretics with distinct mechanisms of action (bumetanide, furosemide, and amiloride) could modulate ocular growth responses during refractive development. To determine whether any observed effects were dependent on imposed optical defocus, ocular responses to negative and positive lens-induced defocus were compared with a No Lens condition, enabling assessment of diuretic effects on ocular growth in the absence of refractive blur. Further, we aimed to characterize the effects of diuretic-mediated perturbation of retinal ion transport on light-evoked retinal function. Specifically, electroretinography was used to assess whether pharmacological inhibition of ion cotransporters alters retinal ON and OFF pathway responses and outer retinal activity under baseline (No Lens) conditions, both acutely following intravitreal injection and after the 4-day rearing period. This approach was employed to determine whether diuretic-induced modulation of retinal light processing was apparent in the short and longer term, and whether there were potential negative impacts on retinal function following application of such drugs.

Based on the RIDE model and prior evidence linking retinal ion transport to light-dependent fluid dynamics, we hypothesized that inhibition of ion-driven transretinal and RPE fluid efflux would alter refractive compensation to optical defocus, while having minimal effects on refraction and ocular growth under No Lens conditions. Furthermore, we predicted that diuretic-induced modulation of refractive state would be accompanied by alterations in light-evoked retinal function, reflected in changes to electroretinographic ON and OFF pathway responses and outer retinal activity.

## 2 Materials and methods

### 2.1 Ethics statement

All procedures were conducted in accordance with La Trobe University Animal Ethics Committee guidelines (Approval No. 08/30P) and adhere to the European Communities Council Directive of 24 November 1986 (86/609/EEC) and the ARVO Statement for the use of Animals in Ophthalmic and Vision Research. Chick wellbeing and cleanliness of lenses were monitored twice daily for the duration of the rearing period; all protocols were designed to minimize distress in animals and all surgical procedures were performed under Ketamine/Xylazine anesthetic.

### 2.2 Animals and rearing

A total of 169 male hatchling chicks (Leghorn/Australorp) obtained from a local hatchery were raised from day 0–9 under a 12/12 h day/night cycle in a light and temperature controlled (30 ± 0.5°C) enclosure. Ambient illuminance was maintained constantly at 183 lux during the 12 h day of a normal diurnal (ND) light cycle via a 20W halogen light globe in the roof of the enclosure. Experimental procedures were not initiated until day 5 to allow time for myelination of the optic nerve of the left eye to become equivalent to that of the right (70, 71). Male chicks were utilized to minimize the potential impact of neuroendocrine confounding influences on drug interactions with ion channel function. The number of animals included in each condition is presented in Supplementary Table 1.

On day 5 post-hatching, chicks were anesthetized (in the middle of the day cycle) with a Ketamine 45 mg/kg: Xylazine 4.5 mg/kg intramuscular injection and the right (experimental) eyes [EE] were intravitreally injected with either 5 μL of (dimethyl sulfoxide (DMSO) alone or one of 1 mM bumetanide, furosemide or amiloride in DMSO carrier solution resulting in a 5 × 10^–6^M effective concentration of each diuretic (Sigma-Aldrich, St. Louis, MO). Drug dosages were selected based on a large literature referenced above examining the effect of these compounds on retinal and RPE fluid regulation (8, 12, 14, 34, 41, 42, 63, 72–75), and following our own initial investigations (32). DMSO was used as the primary carrier rather than saline as amiloride and bumetanide do not dissolve well in PBS whereas all 3 diuretics will dissolve in DMSO. Fellow left eyes (FE) were injected intravitreally with 5μl of DMSO to control for biological effects of the 5 μL increase in intravitreal volume following injection of drugs in the DMSO carrier solution in the EE.

Following intravitreal injections, a monocular defocusing goggle (+ 10 D or -10 D) made from modified human polymethyl methacrylate (PMMA) hard contact lenses (8.1 mm diameter; Australian Custom Lenses, Victoria, Australia) affixed to a complementary 22 mm Velcro hook fastener ring to the periocular feathers for 4 days. The + 10 D or -10 D optical defocus conditions were expected to induce hyperopia and myopia, respectively. A No Lens control group was also utilized to assess the effects of the diuretics on the eye without the presence of blur.

### 2.3 Biometric analysis

On day 9, by which time maximal refractive compensation should have occurred in non-drug treated lensed animals (76), chicks were anesthetized (ketamine 45 mg/kg: xylazine 4.5 mg/kg) and eyes were refracted with retinoscopy (Keeler, Vista Diagnostic Instruments). A-Scan ultrasonography (A-Scan III, TSL: Teknar, Inc., St Louis, 7 MHz probe) was used to measure an average of at least 3 scans for axial dimensions.

### 2.4 Electrophysiology to ascertain effects of diuretics on functional vision

To better understand the impact of the experimental drugs on retinal function, the ERGs of No Lens chicks (*N* = 3–4 per group) were measured at 0 and 96 h following single intravitreal injection of one of the three drugs or carrier alone at the same volume and concentration described above.

A square wave 500 ms light ON, 500 ms light OFF stimulation protocol (150 mm ganzfeld stimulator with peak luminance 50 cd/m^2^ (Tektronix J6523 narrow angle luminance probe) was employed such that retinal On and Off responses could be separately observed. An intravitreal electrode (Ag/AgCl) was inserted under ketamine/xylazine anesthesia (45 mg/kg: Xylazine 4.5 mg/kg i.m.) via a catheter placement unit, with scleral reference. Signals were recorded in dark-adapted animals (under maintained anesthesia) via a Powerlab amplifier (ADI, Sydney, Australia) and band-pass filtered (0.3–1,000 Hz). Twenty potentials were averaged in each run, and 5 such runs were recorded for each eye. Graphs were generated using IGOR Pro 6.22 for Mac.

The ERG protocol was designed to assess the following components: the *a*-*b*-wave complex (photoreceptor, ON bipolar and Müller cell response to light onset), and the *d*-wave (photoreceptor and OFF bipolar response to light offset) (12, 77–83).

### 2.5 Data analysis

Biometric data utilized in the preceding analyses consist of the difference of the fellow eye subtracted from the experimental eye for the variables refractive state, axial length, vitreous chamber depth and anterior chamber depth. The resultant value for each animal was totaled and averaged for each experimental condition to obtain a measure of mean difference for each dependant variable to control for within subject effects such as small growth variations in the overall size of the chicks. Following exclusion of outliers > 1.5 times the boxplot interquartile range, difference measures were analyzed via a series of 2 (3 Lens × 4 Drug) way Analysis of Variance (ANOVA) with significance criterion of alpha = 0.05 using JASP (84). Significant main effects were examined using either Tukey or Games-Howell (GH) *post-hoc* testing, and interaction effects were further explored through Simple Main Effects Analysis, followed by either Tukey or GH *post-hoc* testing when appropriate.

To examine relationships between refractive state and ocular growth measures, Pearson correlation coefficients were calculated between refractive error, axial length, vitreous chamber depth, and anterior chamber depth. Correlations were first assessed across the full dataset to characterize overall associations among growth and refraction. Then, to determine whether these associations were driven by imposed blur, partial correlation analyses were conducted, controlling for lens condition. This approach allowed assessment of the relationships between refraction and growth independent of lens-induced effects.

For ERGs, recordings obtained from No Lens chicks were averaged within each drug condition (amiloride, furosemide, bumetanide, and DMSO carrier) at the acute (0 h) and delayed (96 h) time points. Grand mean waveforms (± SE) were computed across animals following screening for waveform artifacts. Amplitudes were quantified for the a-, b-, and d-waves to assess outer retinal photoreceptor activity, ON and OFF pathway activation, respectively. As described above, long-flash stimuli were used to dissociate ON and OFF components of the ERG, enabling assessment of alterations in retinal signaling induced by pharmacological manipulation of ion cotransporters. Comparisons across drug conditions and time points were used to determine whether diuretic treatment produced acute or sustained alterations in retinal light responses independent of imposed optical defocus. ERG analyses were exploratory in nature and were intended to provide functional insight.

## 3 Results

### 3.1 Effect of lens and diuretics on refractive error and ocular growth

#### 3.1.1 Refractive error development is modulated by diuretics

**FIGURE 2:**
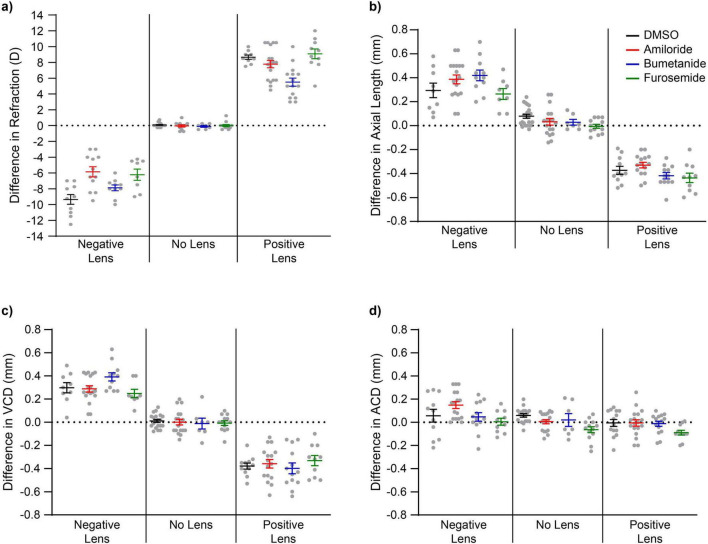
Effects of diuretic treatment on refractive compensation and ocular growth under lens-induced defocus. Mean ± SEM for **(a)** refractive state, **(b)** axial length, **(c)** vitreous chamber depth, and **(d)** anterior chamber depth across lens conditions (-10 D, + 10 D, no lens) and drug treatments (DMSO, amiloride, bumetanide, furosemide) at 4 days post-injection (day 9 post-hatching). Negative lens conditions induced myopic compensation and positive lens conditions induced hyperopic compensation in control (DMSO) eyes. Diuretic treatment differentially altered these responses: amiloride and furosemide suppressed myopic compensation under -10 D lenses, whereas bumetanide most strongly inhibited hyperopic compensation under + 10 D lenses. Axial length changes broadly paralleled refractive outcomes, with modest drug-dependent modulation. In contrast, vitreous chamber depth was primarily driven by lens condition, with minimal drug effects. Anterior chamber depth was significantly influenced by drug treatment independent of lens condition, with amiloride increasing and furosemide decreasing anterior chamber depth relative to controls.

By 4 days post-injection (day 9 post-hatching) each of the diuretic drugs had altered the typically reported growth response to lens-induced defocus. In general, the three diuretics suppressed refractive compensation to -10D lenses, with amiloride showing the greatest degree of suppression. Bumetanide inhibited refractive compensation to + 10D lenses to the greatest extent (Figure 2a and Supplementary Table 1). Consistent with these observations, two-way ANOVA revealed a significant main effect of Lens condition on refractive state (*F*_2_, _131_ = 1058.32, *p* < 0.001, η*_*p*_*^2^ = 0.942), a significant main effect of Drug treatment (*F*_3_, _131_ = 8.56, *p* < 0.001, η*_*p*_*^2^ = 0.164), and a significant Lens × Drug interaction (*F*_6_, _131_ = 7.35, *p* < 0.001, η*_*p*_*^2^ = 0.252), indicating that the effect of diuretics on refraction was strongly dependent on the sign of imposed defocus. *Post hoc* analyses showed that under negative lens conditions, both furosemide and amiloride significantly suppressed myopia relative to DMSO controls, whereas refractive error for bumetanide did not differ from DMSO. In contrast, under positive lens conditions, bumetanide-treated eyes were significantly less hyperopic than all other drug groups, and amiloride-treated eyes were also less hyperopic than DMSO controls, suggesting partial reduction of hyperopic compensation with this drug. No significant differences in refractive state were observed between drug groups in the No Lens condition, demonstrating that diuretic treatment did not alter baseline refraction in the absence of imposed defocus.

#### 3.1.2 Axial length and vitreous chamber depth show moderate impact of diuretic drugs on growth

Means data showed that measures of axial and vitreous chamber elongation were largely consistent with changes in refractive state (Figures 2b,). Assessment of overall ocular dimensions via axial length showed a significant main effect of Lens (*F*_2_, _142_ = 447.45, *p* < 0.001, η*_*p*_*^2^ = 0.863) and of Drug (*F*_3_, _142_ = 3.75, *p* = 0.012, η*_*p*_*^2^ = 0.073). The Lens × Drug interaction approached statistical significance with a medium effect size (*F*_6_, _142_ = 2.07, *p* = 0.060, η*_*p*_*^2^ = 0.080) (85), suggesting a modest, lens-dependent modulation of drug effects. Simple main effects analyses indicated significant differences in axial length between all lens groups, and for drug groups the amiloride-, and furosemide-treated eyes differed significantly from one another whereby overall, amiloride-treated eyes were longest and furosemide-treated eyes were shortest. These findings indicate that diuretics exert measurable effects on axial length, although the magnitude of these effects varies and is only weakly dependent on the sign of optical defocus.

Vitreous chamber depth (Figure 2c) reflected expected changes associated with imposed optical defocus, indicated by a significant main effect of Lens (*F*_2_, _142_ = 384.21, *p* < 0.001, partial η^2^ = 0.844). Neither the main effect of Drug (*F*_2_, _142_ = 0.23, *p* = 0.873, partial η^2^ = 0.005) nor the Lens × Drug interaction (*F*_2_, _142_ = 1.70, *p* = 0.125, partial η^2^ = 0.067) reached significance, with small and medium effect sizes, respectively, indicating that posterior ocular growth was driven primarily by optical defocus rather than pharmacological manipulation.

#### 3.1.3 Anterior chamber depth

Interestingly, anterior chamber depth was significantly affected by both Lens condition (*F*_2_, _153_ = 9.58, *p* < 0.001, partial η^2^ = 0.111) and Drug treatment (*F*_2_, _153_ = 6.24, *p* < 0.001, partial η^2^ = 0.109), with no significant Lens × Drug interaction (*F*_2_, _153_ = 1.42, *p* = 0.209, partial η^2^ = 0.053). These results indicate consistent drug effects across lens conditions. *Post hoc* analyses showed that amiloride was generally associated with increased anterior chamber depth, whereas furosemide was associated with a reduction in anterior chamber depth relative to DMSO controls (Figure 2d). These findings suggest that anterior segment growth is sensitive to pharmacological manipulation of ion transport mechanisms, independent of lens-induced refractive compensation. Tables 1, 2 summarize the main statistical outcomes in context with lens and drug targets for all biometric measures.

**TABLE 1:** Summary of main and interaction effects for ANOVA for refractive state, axial length, vitreous chamber depth and anterior chamber depth.

	Refractive state	Axial length	Vitreous chamber depth	Anterior chamber depth
Lens	*F* = 1058.315, *p* < 0.001	*F* = 447.447, *p* < 0.001	*F* = 384.206, *p* < 0.001	*F* = 9.578, *p* < 0.001
	Partial η^2^ = 0.942	Partial η^2^ = 0.863	Partial η^2^ = 0.844	Partial η^2^ = 0.111
Drug	*F* = 8.558, *p* < 0.001	*F* = 3.754, *p* = 0.012	*F* = 0.233, *p* = 0.873	*F* = 6.243, *p* < 0.001
	Partial η^2^ = 0.164	Partial η^2^ = 0.073	Partial η^2^ = 0.005	Partial η^2^ = 0.109
Lens × drug	*F* = 7.349, *p* < 0.001	*F* = 2.072, *p* = 0.060	*F* = 1.702, *p* = 0.125	*F* = 1.423, *p* = 0.209
	Partial η^2^ = 0.252	Partial η^2^ = 0.080	Partial η^2^ = 0.067	Partial η^2^ = 0.053

**TABLE 2:** Summary of main and interaction effects of lens condition and diuretic treatment on refractive state and ocular growth.

Outcome measure	Main effect: lens	Main effect: drug	Lens × drug interaction	*Post hoc* summary	Drug target summary
Refractive state	Significant (strong effect)	Significant (moderate effect)	Significant	-10D lenses: Amiloride (ENaC) and furosemide suppressed myopic compensation vs. DMSO; bumetanide = no difference from control. + 10D lenses: Bumetanide (NKCC) produced the greatest inhibition of hyperopic compensation (less hyperopic than all groups); amiloride (ENaC) also reduced hyperopia vs. DMSO. No lens: No differences between drug groups	ENaC and KCC targets = decreased myopia NKCC = decreased hyperopia
Axial length	Significant (strong effect)	Significant (small/moderate effect)	Not significant (medium effect)	All lens groups differed from each other. Amiloride eyes were longest, furosemide (KCC) eyes shortest. Modest drug modulation of growth, weakly dependent on defocus sign.	ENaC = longest eyes KCC = shortest eyes
Vitreous chamber depth	Significant (strong effect)	Not significant	Not significant	Growth primarily driven by lens condition. No meaningful differences between drug groups.	
Anterior chamber depth	Significant (moderate effect)	Significant (moderate effect)	Not significant	Amiloride (ENaC)increased anterior chamber depth vs. DMSO. Furosemide (KCC) decreased anterior chamber depth vs. DMSO. Effects consistent across lens conditions (no interaction).	ENaC = deeper anterior vs. control drug KCC = shallower anterior vs. control drug

### 3.2 Relationships between refractive error and ocular dimensions

**FIGURE 3:**
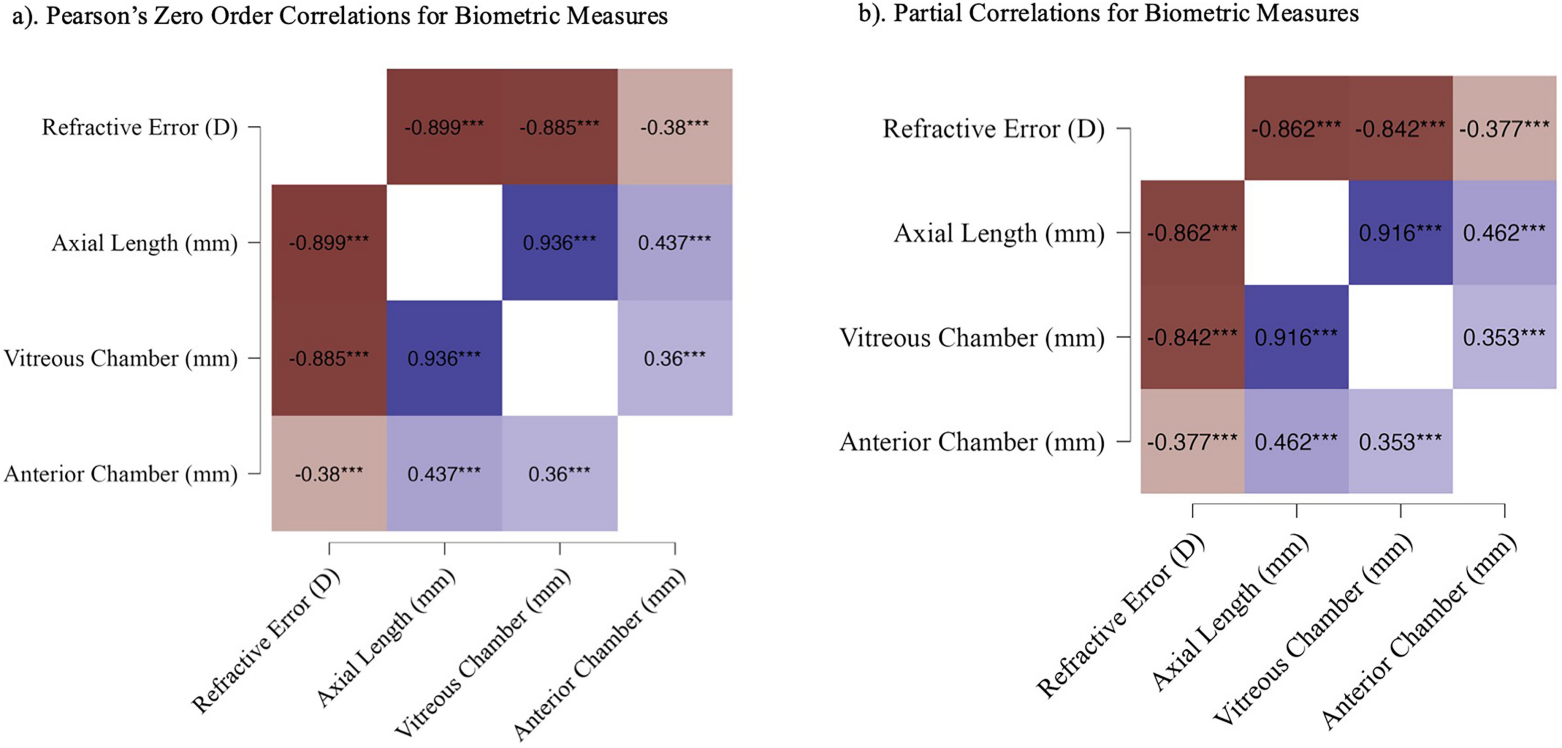
Heat maps depicting. **(a)** Zero-order correlation coefficients for refractive error, axial length, vitreous chamber depth and anterior chamber depth across all conditions, and **(b)** partial correlations for refractive error, axial length, vitreous chamber depth and anterior chamber depth controlling for lens condition (-10 D, + 10 D, or no lens). Reder colors indicate stronger negative correlations and bluer colors indicate stronger positive correlations, with color intensity reflecting correlation magnitude. ***p < 0.0001.

Pearson correlation analyses examining general relationships between biometric measures revealed strong associations between refractive state, overall axial length and posterior ocular dimensions (Figure 3a). Refractive error was highly correlated with axial length and vitreous chamber depth, consistent with axial elongation as the primary structural correlate of refractive shifts. Axial length and vitreous chamber depth were themselves strongly correlated. Correlations between refractive error and anterior chamber depth were weaker, though still significant, indicating a secondary contribution of anterior growth to refractive state. Anterior chamber depth was modestly correlated with both axial length and vitreous chamber depth. Partial correlation analyses controlling for lens condition conducted to determine whether these relationships were driven by imposed optical defocus revealed a similar pattern of results consistent with anterior chamber depth making a lesser contribution to refractive compensation than vitreous chamber depth and axial length (Figure 3b). These findings indicate that diuretics do not disrupt the expected association between refractive error and posterior growth.

### 3.3 Electrophysiological assessment of diuretics on outer retinal function

**FIGURE 4:**
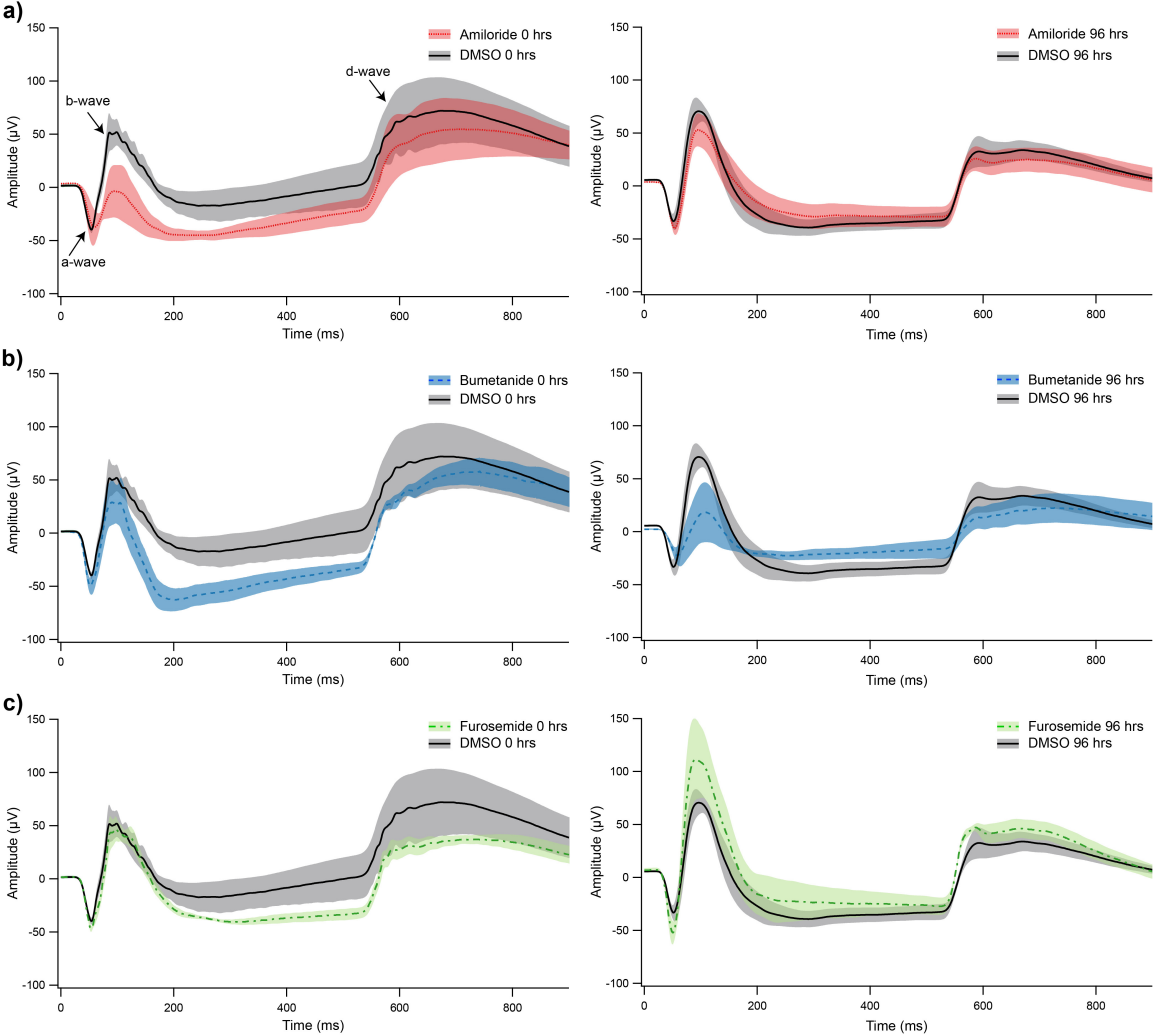
Mean electroretinograms (ERGs) with Standard Error fringes recorded at 0 and 96 h after single injection of amiloride **(a)**, bumetanide **(b)**, furosemide **(c)**, each compared with DMSO (recorded at either 0 or 96 h post-injection).

In order to monitor the effect on outer retinal function of the carrier solution DMSO and the three diuretic agents carried in DMSO, both acutely and at the end of rearing, electroretinograms were recorded from separate groups of No Lens chicks at 0 and 96 h post-injection. The mean (± SE) of 100 recordings and the associated variation for all three drugs compared to DMSO at 0 and 96 h post-injection are shown in Figure 4 (shading indicates SE).

ERGs revealed the expected a-/b-wave complex in response to onset of the flash stimulus, and d-wave at stimulus offset in all conditions. Mean waveforms revealed reduction of the b-wave in the 0-hour amiloride and 96-hour bumetanide conditions, relative to time-matched DMSO controls (Figure 4 and Table 3). The b-wave was large, relative to controls, in the 96-h furosemide group. Consistent with these observations, two-way ANOVA revealed a significant main effect of Drug on the b-wave (*F*_3_, _19_ = 4.17, *p* = 0.020, partial η^2^ = 0.397). *Post hoc* analysis demonstrated that the b-wave was significantly smaller in both amiloride and bumetanide conditions relative to the furosemide condition. No significant effects of Drug, or Drug and Time interactions, were observed for the a-wave or d-wave amplitude. These changes in the b-wave amplitude affected the b/d-wave ratio (an index of the relative strength of ON versus OFF pathway signaling), with amiloride and bumetanide chicks displaying smaller ratios relative to age-matched DMSO controls at 0- and 96-h, respectively. By contrast, furosemide chicks at 96-h displayed very large b/d ratios relative to DMSO controls (Table 3).

**TABLE 3:** Mean ERG waveform amplitude and ratios for recordings 0 and 96 h post intravitreal injection of amiloride, bumetanide, furosemide or DMSO in No Lens chicks.

Time post-injection	Drug	N	Mean *a* wave amplitude (μ V)	Mean *b* wave amplitude (μ V)	Mean *d* wave amplitude (μ V)	*a/b* wave ratio	*b/d* wave ratio
0 h	Amiloride	3	51.53	44.06	80.49	1.67	0.60
	Bumetanide	4	53.68	89.99	93.07	0.72	1.13
	Furosemide	4	49.32	103.38	76.09	0.50	1.46
	DMSO	3	40.41	97.41	73.00	0.44	1.43
96 h	Amiloride	3	40.79	93.81	61.7	0.46	1.59
	Bumetanide	3	25.68	44.47	41.9	1.05	1.07
	Furosemide	3	54.42	173.90	77.73	0.32	2.28
	DMSO	4	36.90	115.05	72.21	0.326	1.64

## 4 Discussion

The present findings demonstrate that single-dose intravitreal injection of common diuretic agents alters refractive compensation through action on retinal and RPE ion transport mechanisms regulating retinal/RPE Na^+^, K^+^, and Cl^–^ fluxes, with consequent alterations in ERG responses that reflect changes in retinal ion dynamics and associated neurotransmission. Thus, it provides evidence for a retinal locus through which diuretic-sensitive ion transport influence refractive compensation through modification of the rate of vitreous-to-choroidal fluid exchange via regulation of ion co-transport systems and osmotic gradients to drive ocular growth.

Relative to DMSO control, 4 days of selective inhibition of either the NKCC1 or the KCC2 symporter, or the Na^+^/H^+^ exchanger by diuretics bumetanide, furosemide or amiloride, respectively, reduced the degree of myopia in young chicks reared with -10 D lenses. The drugs also showed some inhibitory effects on compensation to + 10 D defocus, consistent with a Lens × Drug interaction observed in refraction measures.

The study extends earlier empirical findings of Crewther et al. (32) and theoretical predictions of the RIDE model (21) by demonstrating modulation of refractive state and ocular growth following disruption of ion co-transport systems. It is further strengthened by recent elemental and ultrastructural analyses demonstrating that the retina/RPE/choroid complex maintains stable, spatially organized gradients of Na^+^, K^+^, and Cl^–^ that generate osmotic forces driving continuous fluid movement from vitreous to choroid along gradients critically dependent on the coordinated activity of Na^+^/K^+^ pumps, NKCC and KCC cotransporters, and associated glial cells (86–88), with such gradients showing rapid ionic and gene-related modification under conditions of blur (27, 29, 89, 90). Together, these findings indicate that that administration of diuretic agents may be effective in reducing the impact of defocus-induced growth that would normally lead to more severe myopia and associated pathologies (4, 91, 92). As current therapies for the management of myopia, including spectacle correction or corneal laser surgery, do not reduce the risk of associated pathology, translation of these findings to mammalian models, and ultimately human myopia, is essential.

### 4.1 Diuretic modulation of retinal ion transport in refractive compensation to defocus

Both amiloride and furosemide are less specific in their protein targets compared to bumetanide. Amiloride inhibits epithelial sodium channels and Na^+^/H^+^ exchangers, processes that are fundamental to intracellular pH regulation, osmotic balance, and transepithelial water movement (33, 93–95). Bumetanide and furosemide inhibit Na^+^-K^+^-2Cl^–^ cotransporters, with bumetanide showing greater selectivity for NKCC1, whereas furosemide additionally affects KCC subtypes expressed by bipolar cells and NKCC receptors on photoreceptors (42, 60, 65, 96–100) and, in a concentration dependent manner, photoreceptor and bipolar cell chloride conductances.

In the current study, amiloride led to a general inhibition of compensation to either sign of defocus after 4 days of rearing, suggesting that this drug, through its action on Na^+^-dependent transport systems on both the apical membrane of the RPE and the ciliary epithelium, impacts ocular cellular homeostasis in a global manner. Such disruption is expected to influence Müller cell-mediated buffering of extracellular Na^+^, K^+^, and Cl^–^, with secondary consequences for neuronal excitability and fluid regulation, potentially affecting mitochondrial function and the energetic capacity to sustain retinal signaling (16, 95, 101, 102).

Furosemide also significantly decreased myopic compensation. In line with disruption of osmotic gradients that normally drive fluid efflux from the vitreous chamber via KCC- and NKCC-mediated Cl^–^ transport across retinal neurons, Müller cells, and the RPE, furosemide significantly reduced refractive compensation to negative lens defocus and resulted in modest changes in axial length and vitreous chamber depth. This suggests that furosemide may interfere with ion-dependent fluid exchange to drive eye growth in response to ocular defocus signals, potentially via more selective Cl^–^-dependent disruption of fluid transport. In contrast, bumetanide exhibited a modest effect on compensation to negative lenses, but significantly reduced hyperopic compensation compared to control, perhaps reflecting an impact of more specific modulation of NKCC co-transport systems compared to furosemide. This reduced effect of bumetanide on refractive compensation to negative optical defocus relative to the previous findings of Crewther et al. (32) may reflect a contribution of the carrier (DMSO vs. saline), where DMSO has potential to alter baseline ionic and osmotic conditions within the retina and RPE due to induced membrane permeability pores enhancing entry of hydrophilic agents and fluid across lipophilic membranes (103–105).

#### 4.1.1 Anterior chamber changes with diuretic treatment

Drug-specific alterations in anterior chamber depth are also consistent with differential effects on ion channel–mediated fluid transport across the eye, suggesting effects of drug delivery impacting fluid dynamics on anterior dimensions. Amiloride was associated with anterior chamber deepening, most evident under negative lens conditions, a pattern consistent with inhibition of epithelial sodium channels and Na^+^/H^+^ exchange pathways that regulate aqueous humor secretion and outflow at the ciliary epithelium and trabecular meshwork, thus shifting intraocular fluid distribution toward the anterior chamber (33, 46, 106). In contrast, furosemide produced shallower anterior chambers, consistent with disruption of Cl^–^-dependent transport mechanisms that contribute to aqueous humor formation and osmotic balance, reducing fluid secretion and thus altering osmotic pressure gradients across anterior structures (33, 107–111). Consistent with Crewther et al. (32), bumetanide produced smaller and variable changes in anterior chamber depth. Thus, the anterior chamber depth is sensitive to diuretic-induced modulation of fluid dynamics, but this is likely to be driven by ion concentration gradients rather than blur signaling *per se*.

### 4.2 ERG waveform changes as functional indicators of altered ionic homeostasis

ERG waveform changes are sensitive to alterations in outer retinal processes and RPE resistivity, with predominant contributions from photoreceptors, bipolar cells, and Müller cells responding to light onset and offset (112). ERG waveform changes are sensitive to alterations in outer retinal processes and RPE resistivity, with predominant contributions from photoreceptors, bipolar cells, and Müller cells responding to light onset and offset (112), and can therefore provide an index of diuretic modulation of retinal ion transport and associated neuron function. Consistent with the expectation that amiloride, furosemide and bumetanide would alter the outer retinal ionic environment that underlies these ERG cellular responses to light, there was a strong effect of drugs on the ERG waveforms. Most notably, amiloride and bumetanide produced a reduction in the b-wave and associated b/d-wave ratio, while furosemide enhanced the b-wave and b/d-wave ratio. Previous studies have similarly reported concentration-dependent and variable modulation of the b-wave following acute administration of furosemide, bumetanide, and amiloride administration (39, 43, 72).

These results add to evidence that an altered retinal ionic microenvironment, mediated potentially via Müller cell potassium exchange, sodium-dependent transporters and downstream RPE fluid movement, may modify the rate of vitreous-to-choroidal fluid efflux and interact with outer retinal responsivity to modify axial elongation and subsequent refractive state. This supports the notion that interactions between ionic regulation, SRS hydration and ON/OFF pathway signaling contributes to sign-dependent ocular growth patterns.

### 4.3 Ion transport, transretinal potential, and fluid flow in refractive compensation: implications for therapeutic modulation of myopia

Collectively, these findings support a model in which retinal ion transport and osmotic regulation play an important role in visually guided eye growth. Diuretics appear to act by altering the ion-driven fluid movements that translate retinal signaling into changes in axial elongation. Future work should now extend examination of the link between cellular responses to the visual environment factors linked to altered eye growth, and ion homeostasis. In particular, Müller glial cells represent a key cellular mechanism for integrating ion transport with neuronal function (16, 17, 22). By regulating extracellular ionic exchange, pH, and fluid flow, Müller cells establish the ionic environment that shapes synaptic gain and neuronal excitability across ON and OFF pathways. Disruption of NKCC, KCC, or Na^+^/H^+^ exchange is predicted to alter Müller cell buffering capacity (22), leading to secondary changes in membrane potential, intracellular chloride levels, and osmotic balance across retinal neurons and the RPE. These ionic shifts are expected to influence calcium-dependent processes indirectly, including glutamatergic transmission at photoreceptor-bipolar synapses and inhibitory GABAergic signaling, thereby modifying ON/OFF pathway balance.

At a systems level, these mechanisms align with evolving models of emmetropization that emphasize ion-driven fluid dynamics. The osmotic gradient framework described by Marshall and Crewther (86, 87) and the temporal ON/OFF modulation model proposed by Crewther et al. (113) both predict that retinal ion transport capacity constrains how visual signals are converted into transretinal potential changes and fluid efflux further strengthens this interpretation. Eye movements across structured visual scenes may generate fast-ON/slow-OFF or fast-OFF/slow-ON temporal asymmetries depending on the sign of defocus, thereby modulating transretinal potential and fluid efflux (113). Diuretic interference with ion transport could thus be expected to blunt these visually driven changes in transretinal potential by limiting the retina’s capacity to sustain ionic gradients, attenuating myopic growth without abolishing emmetropization. Testing how diuretic-induced disruption of ion gradients alters the temporal signal processing will be critical for establishing causal links between ionic homeostasis, retinal signaling, and eye growth as a mechanism for therapeutic control of the abnormal ocular elongation and associated pathologies associated with severe myopia.

Finally, future work should explore how ion-driven changes in retinal signaling interact with neuromodulatory systems implicated in myopia, including dopamine (114, 115) and nitric oxide pathways (29, 116). As these systems are sensitive to changes in neuronal activity and ion transporter dynamics, they may provide insights into how altered retinal ion homeostasis and long-term regulation of ocular growth and myopia prevention.

### 4.4 Limitations

Alternative explanations, including systemic drug effects, retinal adaptation, and changes in retinal impedance, cannot be fully excluded. However, the absence of biometric changes in no-lens eyes, and presence of the effects in between eye difference measures, argues against a purely systemic mechanism, and the preservation of ERG waveform structure argues against non-specific toxicity. ERG measures provide indirect indices of ON and OFF pathway activity and cannot localize effects to specific transporters; thus, further work is needed to explore the link between pharmacological, structural and functional mechanisms of action on refractive compensation following diuretic injection. Although there are slight anatomical and physiological differences between chick and human retinas, the fundamental cellular organization and mechanisms regulating retinal fluid balance and maintenance of retinal ion and water homeostasis in both species occurs through similar ion transporters and aquaporin buffering systems. The diuretic drugs act on these conserved pathways, and as such their core mechanisms of action are expected to operate similarly in chick and human retinal tissue. Thus we argue that the underlying physiological mechanisms affected by diuretic drugs are sufficiently preserved across species to allow meaningful comparison between chick and human retinal responses in future research.

## 5 Conclusion

The present study integrates pharmacological, biometric, and electrophysiological evidence to demonstrate that manipulation of multiple ion transport mechanisms of the retina/RPE resulting in altered fluid dynamics significantly impacts both compensation to lens-induced myopia and lens-induced hyperopia, and leads to drug-specific alterations in ON and OFF pathway function. These findings provide further support for the RIDE Model, implicating reduced ion-driven fluid exchange as an influencing factor in myopia. These notions highlight the need for integrative approaches that move beyond individual ions to examine how coordinated ionic, glial, and neurotransmitter processes shape visually guided eye growth. Thus, targeting ion transport systems through diuretic agents offers an avenue for the therapeutic management of myopia, minimizing the risk of the development of referred ocular pathologies.

## Data Availability

The raw data supporting the conclusions of this article will be made available by the authors, without undue reservation.
