# Supplementary material for: Diuretic inhibition of experimental myopia implicates retinal ion-driven efflux in the regulation of ocular growth
Source: Front Med (Lausanne). 2026 May 11;13:1781955. doi: 10.3389/fmed.2026.1781955 (PMC13199119; doi:10.3389/fmed.2026.1781955)
Supplement: Supplementary file 1 [file Table_1.DOCX]

**Supplementary Table 1**
**Mean (+/- SE) Refractive Error and Axial Length for Experimental Eye vs Fellow Eye across Lens and Drug conditions.**

|  |  | N | EE Ret (D) | FE Ret (D) | EE Axial Length (mm) | FE Axial Length (mm) |
| --- | --- | --- | --- | --- | --- | --- |
| -10D | Amiloride | 18 | -4.87 (0.939) | 0.52 (0.219) | 9.17 (0.062) | 8.78 (0.076) |
|  | Bumetanide | 12 | -5.79 (0.629) | 1.77 (0.450) | 9.25 (0.093) | 8.83 (0.052) |
|  | Furosemide | 9 | -5.01 (1.470) | 0.80 (0.523) | 9.10 (0.112) | 8.83 (0.066) |
|  | DMSO | 11 | -8.60 (0.504) | 0.75 (0.264) | 9.17 (0.111) | 8.78 (0.062) |
| No Lens | Amiloride | 18 | 0.82 (0.306) | 0.66 (0.160) | 8.92 (0.041) | 8.89 (0.061) |
|  | Bumetanide | 8 | 1.75 (0.331) | 1.50 (0.257) | 9.05 (0.123) | 8.95 (0.093) |
|  | Furosemide | 14 | 2.27 (0.373) | 1.84 (0.367) | 8.98 (0.052) | 8.97 (0.047) |
|  | DMSO | 22 | 1.06 (0.261) | 0.80 (0.220) | 8.94 (0.052) | 8.86 (0.052) |
| +10D | Amiloride | 18 | 8.32 (0.668) | 1.07 (0.351) | 8.48 (0.047) | 8.79 (0.046) |
|  | Bumetanide | 14 | 7.75 (0.456) | 2.25 (0.288) | 8.51 (0.057) | 8.94 (0.059) |
|  | Furosemide | 12 | 10.33 (0.665) | 1.33 (0.406) | 8.43 (0.067) | 8.86 (0.047) |
|  | DMSO | 13 | 9.54 (0.507) | 0.71 (0.320) | 8.46 (0.070) | 8.83 (0.067) |

* *Note: EE = Experimental Eye, FE Fellow (control) Eye, Ret (D)= Refractive Error (Dioptres), AL = Axial Length (mm)*
